# Local Skin Inflammation in Cutaneous Leishmaniasis as a Source of Variable Pharmacokinetics and Therapeutic Efficacy of Liposomal Amphotericin B

**DOI:** 10.1128/AAC.00631-18

**Published:** 2018-09-24

**Authors:** Gert-Jan Wijnant, Katrien Van Bocxlaer, Amanda Fortes Francisco, Vanessa Yardley, Andy Harris, Mo Alavijeh, Sudaxshina Murdan, Simon L. Croft

**Affiliations:** a Department of Immunology and Infection, Faculty of Infectious and Tropical Diseases, London School of Hygiene and Tropical Medicine, London, United Kingdom; b Department of Pathogen Molecular Biology, Faculty of Infectious and Tropical Diseases, London School of Hygiene and Tropical Medicine, London, United Kingdom; c Pharmidex Pharmaceutical Services Ltd., London, United Kingdom; d Department of Pharmaceutics, UCL School of Pharmacy, London, United Kingdom

**Keywords:** cutaneous leishmaniasis, inflammation, pharmacokinetics, liposomal amphotericin B

## Abstract

Disfiguring skin lesions caused by several species of the Leishmania parasite characterize cutaneous leishmaniasis (CL). Successful treatment of CL with intravenous (i.v.) liposomal amphotericin B (LAmB) relies on the presence of adequate antibiotic concentrations at the dermal site of infection within the inflamed skin. Here, we have investigated the impact of the local skin inflammation on the pharmacokinetics (PK) and efficacy of LAmB in two murine models of localized CL (Leishmania major and Leishmania mexicana) at three different stages of disease (papule, initial nodule, and established nodule). Twenty-four hours after the administration of one 25 mg/kg of body weight LAmB (i.v.) dose to infected BALB/c mice (*n* = 5), drug accumulation in the skin was found to be dependent on the causative parasite species (L. major > L. mexicana) and the disease stage (papule > initial nodule > established nodule > healthy skin). Elevated tissue drug levels were associated with increased vascular permeability (Evans blue assay) and macrophage infiltration (histomorphometry) in the infected skin, two pathophysiological parameters linked to tissue inflammation. After identical treatment of CL in the two models with 5 × 25 mg/kg LAmB (i.v.), intralesional drug concentrations and reductions in lesion size and parasite load (quantitative PCR [qPCR]) were all ≥2-fold higher for L. major than for L. mexicana. In conclusion, drug penetration of LAmB into CL skin lesions could depend on the disease stage and the causative Leishmania species due to the influence of local tissue inflammation.

## Abstract (precis)

Disfiguring skin lesions caused by several species of the Leishmania parasite characterize cutaneous leishmaniasis (CL). Successful treatment of CL with intravenous (i.v.) liposomal amphotericin B (LAmB) relies on the presence of adequate antibiotic concentrations at the dermal site of infection within the inflamed skin.

## INTRODUCTION

Leishmaniasis is a vector-borne neglected tropical disease caused by over 20 distinct species of the protozoan Leishmania parasite. The two main forms, visceral leishmaniasis (VL) and cutaneous leishmaniasis (CL), continue to pose a major public health problem with significant socioeconomic burden worldwide (1). Current estimates show a global annual incidence of 1 million, 12 million prevalent cases in 98 countries, and over 350 million people at risk of infection (2). CL presents as a wide clinical spectrum of skin syndromes, ranging from severe and rare mucosal leishmaniasis (MCL), diffuse leishmaniasis (DCL) or chronic to the more common, uncomplicated localized leishmaniasis (LCL) lesions. In LCL, a single or limited number of lesions form at the bite site of the parasite-infected female sand fly. A small papule forms, which develops into an initial nodule and then an established nodule with signs of exudation and/or crust formation. The nodule progressively ulcerates and eventually leaves an open wound with raised borders and a crater-like appearance. In most cases, such ulcers slowly self-heal but leave permanent disfiguring scars on the exposed skin areas that are often the cause of serious social stigma (3). Tissue damage and disease in CL are primarily caused by an excessive host immune response against the intracellular infection of dermal macrophages by Leishmania spp. (4). As the dermis fills with a dense and diffuse mixed inflammatory cell infiltrate (including macrophages, lymphocytes, neutrophils, mast cells, and plasma cells), the associated edema drives swelling of the tissue. Epidermal changes (hyperkeratosis, acanthosis, and degeneration of the basal layer), connective tissue damage (collagen lysis), and the formation of noncaseating granuloma can occur (5–9). The immunopathology of LCL shows both similarities (chronic, often ulcerative, dermatosis) and differences (clinical presentation, incubation, and resolution time) among different causative Leishmania species (10, 11). For example, Old World L. major causes so-called “wet” and acute (early ulcerative) CL lesions in the Middle East, seen as large, irregular, and often oozing wounds, which rapidly progress and heal over 2 to 6 months (12, 13). In Central America, New World L. mexicana is the responsible agent for “chiclero's ulcers,” chronic lesions typically found on the ear which spontaneously reepithelize over a period lasting months to even years (14, 15). In a minority of CL cases caused by L. major and L. mexicana, alternative types of skin lesions with different clinical presentations and immune response can develop (12–15).

Treatment of CL is problematic, as long series of painful injections with the toxic pentavalent antimonials remain the standard therapy (16). A better-tolerated but expensive second-line drug requiring intravenous (i.v.) administration and cold chain is AmBisome (LAmB; Gilead, UK) (17). LAmB is a unilamellar liposomal formulation of the polyene antibiotic amphotericin B (AmB), which forms cidal pores in the leishmanial cell membranes by ergosterol binding (18). Several treatment regimens for a total cumulative dose of 20 to 25 mg/kg of body weight are efficacious against CL and MCL (19). However, therapeutic responses vary for the different causative Leishmania species, populations, geographical regions, and clinical settings (20).

We have recently demonstrated that the efficacy of LAmB in murine CL relies on adequate exposure of the active compound AmB at the local site of infection, the skin lesion. Moreover, we also showed higher drug disposition in diseased than in healthy skin (21). Altered pharmacokinetics (PK) at sites of tissue inflammation have been reported previously for antimicrobials (22), anti-inflammatory agents (23), and cancer chemotherapeutics (24). Based on these observations, we formulated three hypotheses, discussed below.

**FIG 1:**
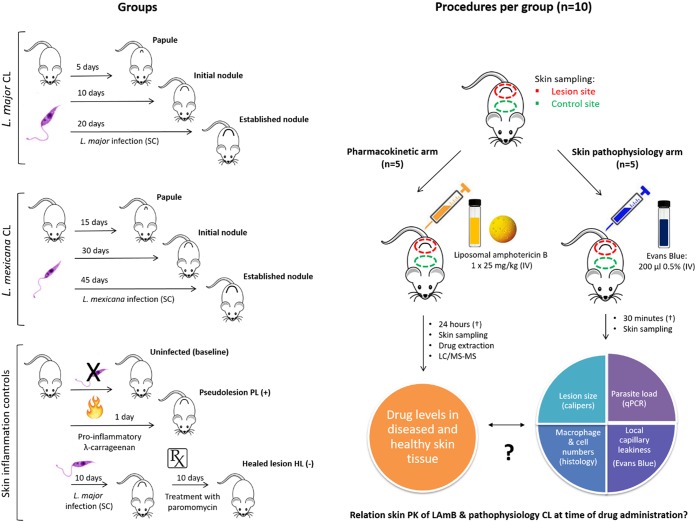
Schematic overview of experimental design to study the influence of skin inflammation in CL on the PK of LAmB.

First, the preferential drug distribution of LAmB in CL lesions over uninfected skin can be explained by the presence and the severity of the local skin inflammation. This could vary among different disease stages of CL and among causative parasite species. In the context of LCL skin inflammation, we have focused only on aspects potentially relevant to the pharmacological action of liposomal drugs. The inflammatory response against the Leishmania infection at the skin inoculation site involves increased vascular permeability and vasodilatation of dermal blood vessels and the infiltration of several types of immune cells, including macrophages, that play a role in tissue swelling and the formation of skin lesions. Second, the underlying mechanisms for altered drug distribution at the inflammatory site are, at least in part, local capillary leakiness (25–28) and influx of drug-loaded macrophages into the skin (29–34). Third, AmB levels accumulating in lesions following LAmB treatment can be a source of variability in treatment outcomes against different Leishmania species. To test the first two hypotheses, we studied the skin PK of LAmB after the administration of a single high dose (1 × 25 mg/kg i.v.), as well as pathophysiological parameters that could influence the drug distribution process from blood to skin using the Evans blue assay (35–37) and histomorphometry. This was done in infected mice and in control mice with various degrees of skin inflammation, as follows: none (uninfected), high (pseudolesion [PL], a new mouse model of local skin inflammation based on the rat paw edema model [38, 39]), or low (healed lesion [HL], cured of CL by paromomycin sulfate [40]). Figure 1 gives an overview of the experimental groups and procedures. To investigate the third hypothesis, we compared intralesional drug accumulation and efficacy in L. major and L. mexicana murine CL following treatment with an identical LAmB dose regimen (5 × 25 mg/kg i.v.).

## RESULTS

### Pharmacokinetic arm: AmB accumulation in skin after LAmB administration.

**FIG 2:**
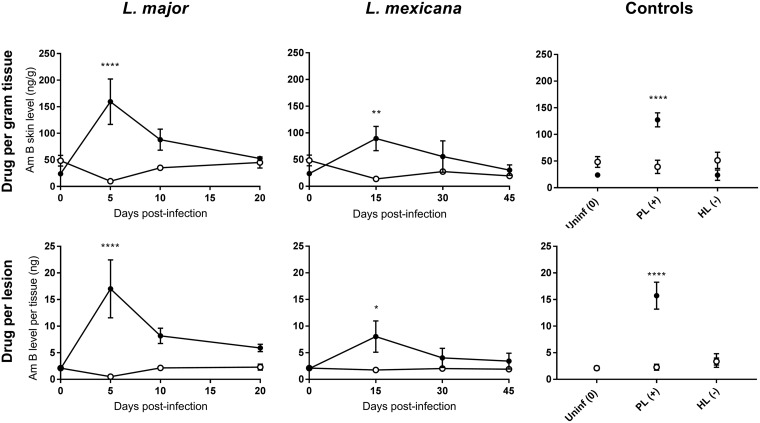
Skin accumulation of amphotericin B (AmB), 24 h after a single intravenous (i.v.) administration of 25 mg/kg AmBisome (LAmB) to CL-infected mice at different time points postinfection and controls. Drug levels were determined in the lesion (●) and healthy control skin (○) site for each animal. CL-infected mice with skin lesions were dosed with LAmB at the time when a papule, an initial nodule, or an established nodule was present on the rump (5, 10, and 20 days after L. major infection, respectively, and 15, 30, and 45 days after L. mexicana infection, respectively). Controls for skin inflammation were uninfected mice (Uninf), pseudolesion (PL; mice with carrageenan-induced inflammatory skin initial nodule), and healed lesion (HL; mice with paromomycin-cured L. major initial nodule). Data are shown as the means ± standard error of the mean (SEM) (*n* = 3 to 5 per group). Statistical analysis was determined with a 2-way ANOVA, followed by Šidák multiple-comparison test. *, *P* < 0.05; **, *P* < 0.01; ***, *P* < 0.001; ****, *P* < 0.0001.

Figure 2 shows AmB accumulation (nanograms of AmB per gram of skin tissue; nanograms of AmB per lesion) in infected and healthy control skin at different stages of murine L. major or L. mexicana CL (papule, initial nodule, and established nodule) 24 h after the administration of a single dose of 25 mg/kg LAmB (i.v.). The morphology of the lesions is shown in Fig. 6a. Table 1 shows AmB lesion-to-healthy-skin ratios, the ratio of the AmB skin level in the lesion over the AmB skin levels in the healthy control skin (calculated from values in Fig. 2, row 1). The ratios indicate that there is a 3-fold decrease in intralesional AmB accumulation when LAmB is administered at late (i.e., established nodule) compared to early (i.e., papule) stages of both L. major and L. mexicana CL. Drug levels were higher in L. major than in L. mexicana lesions at all stages of disease. The disposition of AmB in the PL was significantly higher than in healthy skin (*P* < 0.0001). In contrast, AmB accumulation in HL is not significantly different from that in healthy control skin (*P* = 0.37) and is similar to the baseline levels in uninfected mice. Drug distribution patterns are highly comparable when AmB concentrations are expressed as relative (normalized, in nanograms per gram) or absolute (nanograms per lesion). This indicates that the altered PK of LAmB at different stages of CL is not a consequence of bias introduced by change in tissue volume/weight over the course of infection.

**TABLE 1:** Lesion-to-healthy-skin ratios, based on the values found in lesions (rump) and healthy control skin (back) for the variables AmB accumulation, blood vessel permeability, total number of cells, and number of macrophages^*a*^

Variable	Lesion-to-healthy-skin ratio								
	L. major CL			L. mexicana CL			Controls		
	Papule	Initial nodule	Established nodule	Papule	Initial nodule	Established nodule	Uninf	PL (+)	HL (−)
AmB accumulation	16.2	2.5	1.2	3.7	2	1.6	0.5	3.2	0.5
Blood vessel permeability	5.9	9.4	6.8	2.6	12.5	9.5	1.7	11.7	1.2
No. of cells	1.8	2.3	2.4	1.2	1.2	1.4	1	1.6	1.1
No. of macrophages	5.4	7.2	5.1	3	4.8	4.9	0.9	1.5	4.8

a Data are derived from Fig. 2, 4, and 5.

### Skin pathophysiology arm: factors affecting the PK of LAmB.

**FIG 3:**
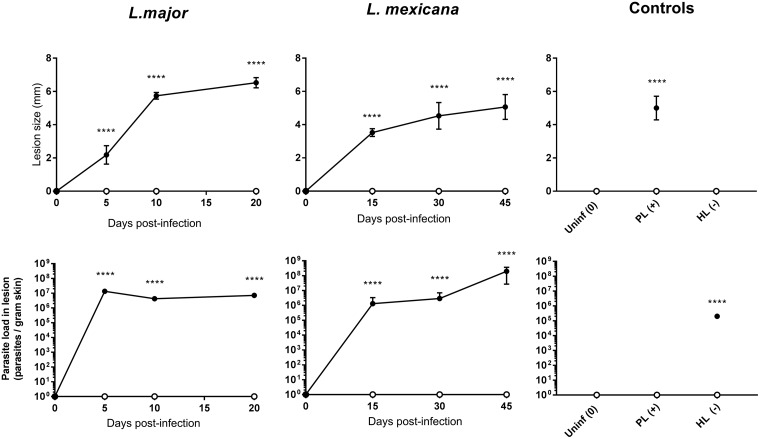
Lesion size (top row) and parasite load (bottom row) into CL-infected mice at different time points postinfection and controls. Lesion size (in millimeters) and parasite load (parasites per gram skin) were determined in the lesion (●) and healthy control skin (○) for each animal. CL-infected mice with skin lesions were measured at the time when a papule, an initial nodule, or an established nodule was present on the rump (5, 10, and 20 days after L. major infection, respectively, and 15, 30, and 45 days after L. mexicana infection, respectively). Controls for skin inflammation were uninfected mice (Uninf), pseudolesion (PL; mice with carrageenan-induced inflammatory skin initial nodule), and healed lesion (HL; mice with paromomycin-cured L. major initial nodule). Data are shown as the means ± SEM (*n* = 3 to 5 per group). Statistical analysis was determined with a 2-way ANOVA, followed by Šidák multiple-comparison test. *, *P* < 0.05; **, *P* < 0.01; ***, *P* < 0.001; ****, *P* < 0.0001.

(i) Lesion characterization: size and parasite load. Fig. 3 shows the lesion characteristics (top row, lesion size; bottom row, parasite load) at different stages of infection by L. major or L. mexicana CL (papule, initial nodule, and established nodule). The morphology of the lesions can be seen in Fig. 6 (a images). L. major lesions increased in size at a more rapid pace than L. mexicana, with different parasite load dynamics over time. During the 20 days following infection with L. major, lesion size gradually increased from 0 to around 7 mm, and parasite load remained stable from day 5. Following infection with L. mexicana, smaller lesions formed (up to 5 mm), and the parasite load gradually increased. The PL swelling of rump skin had a size comparable to that of CL lesions, but as expected, no parasites could be detected in this Leishmania-free type of skin inflammation. In contrast, the HL (day 20, after 10-day treatment with paromomycin) had a lesion size of 0 ± 0 mm, and the parasite load was around 100-fold lower than in the untreated L. major established nodules (day 20). As expected, neither lesion size nor parasite load was measurable in uninfected mice.

### (ii) Evans blue and leakiness of dermal capillaries.

**FIG 4:**
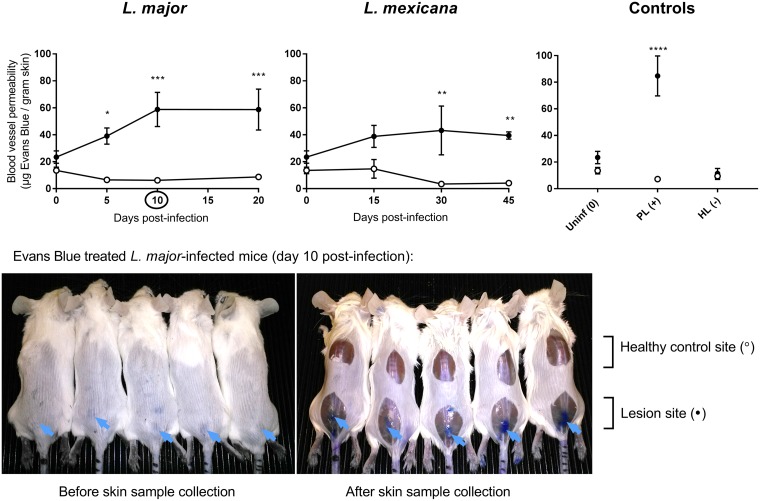
Leakiness of the blood vessels in the skin of CL-infected mice at different time points postinfection and controls. After administration of Evans blue (200 μl 0.5% i.v.), the amount of the blue dye per gram of tissue was determined in the lesion (●) and healthy control skin (○) for all animals. CL-infected mice with skin lesions were dosed with Evans blue at the time when a papule, an initial nodule, or an established nodule was present on the rump (5, 10, and 20 days after L. major infection, respectively, and 15, 30, and 45 days after L. mexicana infection, respectively). Controls for skin inflammation were uninfected mice (Uninf), pseudolesion (PL; mice with carrageenan-induced inflammatory skin initial nodule), and healed lesion (HL; mice with paromomycin-cured L. major initial nodule). Data are shown as the means ± SEM (*n* = 3 to 5 per group). Statistical analysis was determined with a 2-way ANOVA, followed by Šidák multiple-comparison test. *, *P* < 0.05; **, *P* < 0.01; ***, *P* < 0.001; ****, *P* < 0.0001. The picture shows L. major-infected mice (day 10) after 30 min after administration of Evans blue (i.v.). The arrows point at the blue coloration of the CL lesions (before skin sample collection, left photo) as well as intense blue staining of the underlying thoracolumbar fascia (after skin sample collection, right photo).

Fig. 4 shows vascular permeability in infected and healthy control skin at different stages of murine L. major or L. mexicana CL (papule, initial nodule, and established nodule), as evaluated by the Evans blue assay. The morphology of the lesions can be seen in Fig. 6a. Table 1 shows Evans blue lesion-to-healthy-skin ratios, the ratio of the Evans blue skin level in the lesion over the Evans blue skin levels in the healthy control skin (calculated from the values in Fig. 4). The ratios for L. major indicate that compared to healthy control skin, vascular permeability is 6-fold higher in papules and 9-fold higher in initial nodules and established nodules. For L. mexicana, there is 3- to 10-fold increase in permeability compared to healthy skin, and the increase is comparable for papules, initial nodules, and established nodules. Blood vessel leakiness was 12-fold higher (*P* < 0.0001) in the PL than in healthy skin. In HL, vascular permeability is not significantly different from that in healthy control skin (*P* = 0.99) and is similar to the baseline levels in uninfected mice. In the photos in Fig. 4, the intense blue coloration of lesions (due to accumulation of the Evans blue dye) provides an additional qualitative confirmation of capillary leakiness at the site of infection. Such a phenomenon is absent in healthy skin tissues.

### (iii) Skin histomorphometry: inflammatory cells and macrophages.

**FIG 5:**
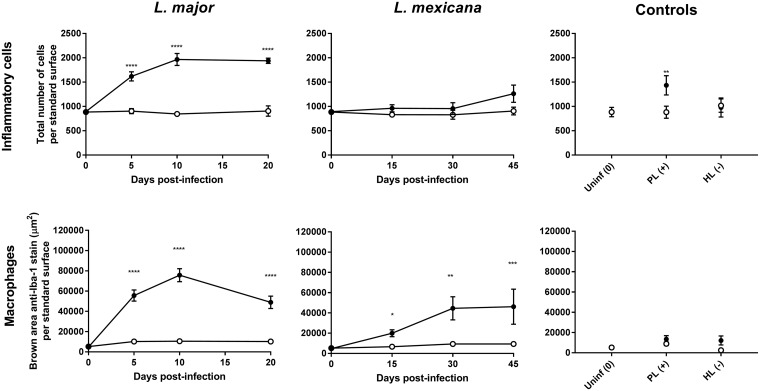
Estimation of the number of cells (top row, H&E stain) and macrophages (bottom row, anti-Iba-1 reaction) at the infected lesion site (rump skin, black bars) and the control site (back skin, white bars) of control mice and CL-infected mice. Measurements in CL-infected mice with skin lesions were performed at the time when a papule, an initial nodule, or an established nodule was present on the rump (5, 10, and 20 days after L. major infection, respectively, and 15, 30, and 45 days after L. mexicana infection, respectively). Controls for skin inflammation were uninfected mice (Uninf), pseudolesion (PL; mice with carrageenan-induced inflammatory skin initial nodule), and healed lesion (HL; mice with paromomycin-cured L. major initial nodule). Standard surface was the picture area showing full skin tissue (epidermis, dermis, and hypodermis) to allow direct comparisons among groups (166,970.7 μm^2^). Data are shown as the means ± SEM (*n* = 3 to 5 per group). Statistical analysis was determined with a 2-way ANOVA, followed by Šidák multiple-comparison test. *, *P* < 0.05; **, *P* < 0.01; ***, *P* < 0.001; ****, *P* < 0.0001.

**FIG 6:**
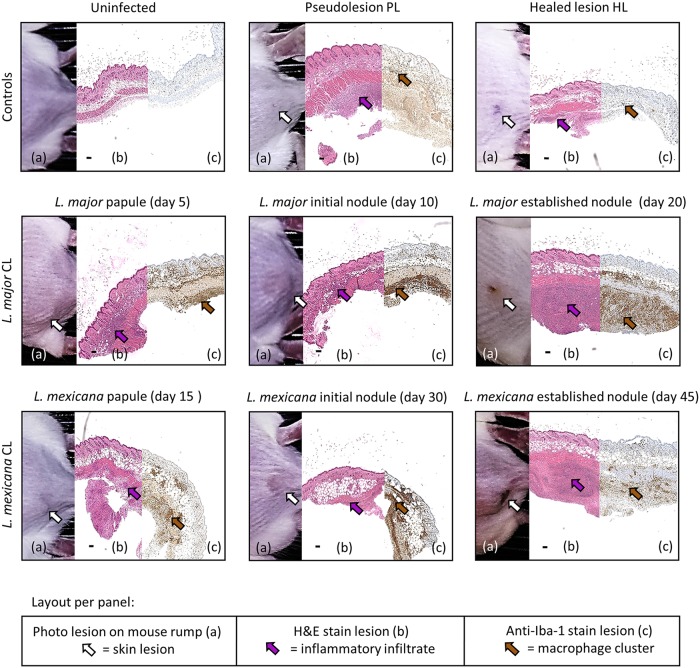
Collage panels of murine skin lesions developed during CL disease progress and controls for skin inflammation. Per panel, photo of the lesion on the rump of the mice (a, white arrow points at lesion), hematoxylin and eosin stain (b, purple arrow points at a cluster of inflammatory cells), and macrophage marker anti-ionized calcium binding adapter molecule 1-antibody stain (c, brown arrow points at a cluster of macrophages). Top row, controls for skin inflammation (uninfected, pseudolesion, and healed lesion). Middle row, L. major CL lesions (papule present at 5 days postinfection, initial nodule present at 10 days postinfection, and an established nodule present at 20 days postinfection). Bottom row, L. mexicana CL lesions (papule present at 15 days postinfection, initial nodule present at 30 days postinfection, and an established nodule present at 45 days postinfection). (b) Black scale bar = 100 μm.

**FIG 7:**
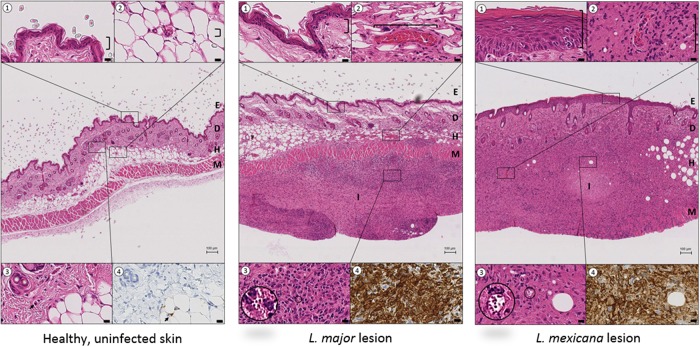
Comparison of mouse skin morphology and macrophage density in healthy, uninfected skin (left), L. major CL lesion (20 days postinfection, middle), and L. mexicana CL lesion (45 days postinfection, right). The central picture in each panel (H&E stain) shows the structural layers of the skin, epidermis (E), dermis (D) and hypodermis (H), with the underlying muscle (M) at ×4 magnification (bar = 100 μm). The insets (1 to 4) highlight details of the central picture (×80 magnification, bar = 10 μm). ①, epidermis; ②, dermal capillaries; ③, Leishmania amastigotes within parasitophorous vacuoles; ④, anti-Iba-1 stain (macrophage marker) of tissue shown in inset ③. In both the L. major and L. mexicana CL lesions, intense inflammatory foci (I) are present in the skin, causing severe disruption of the D and H architecture. Compared to healthy uninfected skin, CL lesions also showed (i) epidermal hyperplasia and acanthosis for L. mexicana but not for L. major (①), (ii) dilated blood vessels, a factor contributing to capillary leakiness (②), and (iii) a large amount of inflammatory cells (③), many of which are macrophages (④).

Fig. 5 shows the number of total cells (top row) and the abundance of macrophages (bottom row) in infected and healthy control skin at different stages of murine L. major or L. mexicana CL (papule, initial nodule, and established nodule). Figure 6 shows the morphology of the lesions (Fig. 6, a images), the hematoxylin and eosin (H&E) stain (Fig. 6, b images), and the anti-Iba-1 stain (Fig. 6, c images). Figure 7 examines the H&E and Iba-1 stains of CL lesions in more detail. Table 1 shows total cell and macrophage lesion-to-healthy-skin ratios, the ratio of the total cell and macrophage skin numbers in the lesion over the total cell and macrophage skin numbers in the healthy control skin (calculated from the values in Fig. 5). The ratios indicate that the number of cells in the tissue double in CL lesions as the disease progresses, and a large fraction of the infiltrated inflammatory cells are macrophages. However, the numbers of inflammatory cells and macrophages in L. major lesions are higher than those in L. mexicana lesions at all stages of disease. In the PL, the number of inflammatory cells was significantly higher than that in healthy skin (*P* = 0.0034), but this was not the case for macrophages specifically (*P* > 0.99). In the HL, the numbers of inflammatory cells and macrophages were not significantly different from those in healthy control skin (*P* > 0.05) and are similar to the baseline levels in uninfected mice.

### (iv) Relationship between PK and pathophysiology parameters.

Table 1 shows the lesion-to-healthy-skin ratios (parameter value in lesion/parameter value in healthy skin) for AmB accumulation (Fig. 2 data, AmB levels in nanograms per gram), blood vessel permeability (Fig. 4 data), number of cells, and number of macrophages (Fig. 5 data). For uninfected mice, the ratios for AmB, blood vessel permeability, cell numbers, and macrophage numbers were around 1, indicating no difference in the values for these parameters between the lesion site (rump skin) and the healthy site (back skin). Comparing Leishmania-infected mice to uninfected mice, AmB accumulation, blood vessel permeability, cell numbers, and macrophage numbers were higher at all three stages of disease for both L. major and L. mexicana. However, these ratios were increased for L. major compared to L. mexicana. The higher ratios for PL than those for uninfected mice indicate increased drug accumulation, as well as blood vessel leakiness, cell numbers, and macrophages in this alternative type of skin inflammation. For HL, however, all lesion-to-healthy skin ratios were highly similar to the baseline ratios found in healthy mice (except for macrophage number). Similar patterns at different stages of disease were found in *L. major*- and L. mexicana-infected mice. A significant increase in ratios for drug accumulation, blood vessel permeability, cell numbers, and macrophage numbers was found in papules (early CL) compared to uninfected mice. In a comparison of the ratios for the papule compared to those for initial nodules and established nodules (later-stage CL), relatively little new additional inflammatory cells and macrophages seemed to infiltrate the skin (for both L. major and L. mexicana), and blood vessel permeability remained stable (for L. major but not L. mexicana).

### Skin PK and efficacy of LAmB in CL.

**FIG 8:**
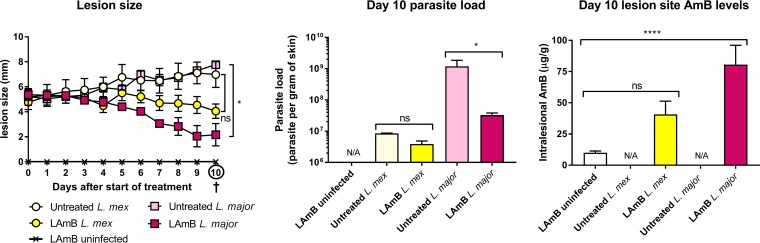
Efficacy and biodistribution of liposomal amphotericin B LAmB) in murine models of L. major and L. mexicana (*L. mex*) CL. Mice were injected (s.c.) with parasite-free medium (uninfected) or infected with L. major or L. mexicana promastigotes in the rump skin. When a nodular lesion had formed at the inoculation site of CL-infected animals (10 and 30 days postinoculation for L. major and L. mexicana, respectively), animals received either 5% dextrose (untreated) or 25 mg/kg LAmB (i.v.) on days 0, 2, 4, 6, and 8. During treatment, lesion size (a) was measured daily. On day 10, lesion skin tissues were collected, and parasite load (b) and AmB levels (c) were determined. Each point represents the mean ± SEM (*n* = 3 to 5 per group). ANOVA (1-way for parasite load and intralesional AmB levels, repeated measures for lesion size), followed by Tukey's multiple-comparison test (*, *P* < 0.05; ****, *P* < 0.0001; ns, not significant) were used. N/A, not applicable.

Finally, we evaluated the efficacy of LAmB against L. major and L. mexicana in the BALB/c mouse model of CL. Figure 8 shows *in vivo* activity and intralesional AmB accumulation on day 10, after treatment of mice with initial nodules with 5 doses of 25 mg/kg LAmB (i.v.) on alternate days (i.e., on days 0, 2, 4, 6, and 8). LAmB showed *in vivo* activity against both CL-causing parasite species. However, reductions in lesion size and parasite load compared to untreated controls were greater than and significant for L. major (*P* = 0.011 and 0.0471) compared to L. mexicana (*P* = 0.25 and 0.99). We also observed almost 2-fold higher AmB levels (in nanograms per gram) in L. major over L. mexicana lesions. In CL-infected skin, drug level concentrations were at least 4-fold higher than those in healthy rump skin of identically uninfected LAmB-treated mice. However, this difference was significant for L. major (*P* < 0.0001) but not for L. mexicana (*P* = 0.15). The L. major data have been reported earlier (21) but are included to enable a direct comparison with L. mexicana (novel data).

## DISCUSSION

Local tissue inflammation in infectious disease can alter the pharmacokinetics (PK) and thus therapeutic outcomes of antimicrobials (41–43). In this work, we have confirmed our hypothesis that the inflamed state of skin lesions in CL alters the PK of LAmB following intravenous drug administration in two mouse models of infection. Our results show that AmB accumulation in CL-infected skin is (i) Leishmania species specific (greater in L. major than in L. mexicana lesions) (ii) disease stage specific (papule > initial nodule > established nodule > healthy skin), and (iii) a plausible cause of the superior *in vivo* efficacy of LAmB against L. major compared to that against L. mexicana.

First, the preferential distribution of LAmB to CL infection sites (L. major > L. mexicana) compared to uninfected ones could be explained by the presence and the severity of the local inflammatory response against the parasites residing in dermal macrophages. Compared to L. mexicana, L. major causes more heavily inflamed (exudative) established nodules with a more rapid, aggressive onset in humans (12–15) and mice (3, 44). Several quantitative biomarkers for skin inflammation in our study confirmed this. The leakiness of the dermal capillaries, swelling/edema in the skin tissue (indicated by lesion size), and numbers of infiltrating macrophages or other inflammatory cells were higher in L. major than in L. mexicana CL at all stages of disease. These findings are consistent with earlier reports (45–47). Moreover, the HL and PL observations support this inflammation-driven theory of enhanced drug accumulation. When the inflammation in L. major-infected skin is largely cleared because of parasite elimination by paromomycin treatment (HL), AmB accumulation, blood vessel permeability, and cell numbers return to baseline levels seen in uninfected skin. However, when inflammation is experimentally induced by injection of λ carrageenan (instead of parasites) in rump skin (similar site to that in CL infection), the local drug concentrations after LAmB administration also increase by over 3-fold. Such a phenomenon could be explained by a 10-fold increase in leakiness of the skin capillaries. The new PL model of local skin inflammation, based on subcutaneous injection of λ carrageenan, could be a useful research tool for dermatoses other than CL, such as skin cancers, atopic dermatitis, or psoriasis (48).

Second, the increased intralesional AmB accumulation after intravenous LAmB dosing of mice with CL in earlier stages of disease (papule > initial nodule > established nodule) could be related to changes in infiltration of phagocytes prone to internalize circulating liposomes and, likely to a lesser degree, capillary leakiness in the dermis. When LAmB is administered to mice with early CL, during the initial massive influx of phagocytes and inflammatory cells into the skin as part of the antileishmanial immune response (4, 11), intralesional drug levels could be increased as AmB-loaded cells migrate from the bloodstream to the infection site. Hence, in later stages of disease, when the number of additional macrophages infiltrating the infected tissue is more limited, skin AmB accumulation could be lower. The known role of phagocyte transport in the delivery of various antibiotics (30–32), including liposomal AmB (41), to local infection sites, as well as our PK and histology data, suggests the plausibility of this hypothesis. Confirmative research should distinguish extra- and intracellular levels in circulating and dermal macrophages after LAmB administration. While phagocytes can increase AmB exposure in the lesion, their therapeutic relevance is still unclear. Cellular lysis, resulting in local release of the drug payload, or impaired parasite survival in these “pretreated” macrophages could play a role. Another pathophysiological factor affecting the PK of LAmB is blood vessel leakiness, a result of vasodilatation and enhanced vascular permeability in the inflamed dermis. Here, we confirmed the existence of this phenomenon in experimental CL for the first time. It could facilitate extravasation of the liposomes (∼80 nm in size) through the dermal capillaries, which under normal physiological conditions have a pore cutoff size of 6 to 12 nm (21). However, it cannot explain a decrease in AmB disposition in lesions as CL progresses by itself, because we found comparable degrees of capillary leakage in papules, initial nodules, and established nodules. Other factors that could affect cellular and dermal PK, such as plasma and tissue protein binding (49), angiogenesis (50), lymphatic drainage, phagocytic capacity, and activation stage of (parasitized) macrophages (33), skin metabolism, clearance by the reticuloendothelial system (51), or the involvement of (nonmacrophage) immune cells, mediators, or responses, were not evaluated in this study. A similar trend of decreasing drug distribution of LAmB to target organs during later disease stages was also found in murine VL (33). However, interestingly, Leishmania-infected livers contain lower rather than higher drug levels than healthy ones.

Third, the *in vivo* activity of LAmB was superior against L. major than against L. mexicana, likely due to inflammation-enhanced and relatively increased drug levels at the infection site. A clear correlation between drug levels of the leishmanicidal, concentration-dependent antibiotic AmB delivered to the lesion and the efficacy of LAmB in murine CL has already been reported (21, 52). Apart from skin PK, there could also be differences in antileishmanial pharmacodynamics (PD) and the resulting PK/PD relationship. An intrinsic species-specific sensitivity to the active compound AmB is unlikely, as *in vitro* 50% effective concentrations (EC_50_s) are comparable (≈0.1 μM) (35). However, the *in vivo* susceptibility could still vary based on the metabolic state of the L. major or L. mexicana parasites in the skin. In chronic lesions with slow disease onset, a quiescent semidormant phenotype of L. mexicana could exist, benefitting its long-term survival and possibly showing reduced drug sensitivity (53–55). Such PK/PD factors could cause variable rate or magnitude of parasite elimination, a combined outcome of drug activity and host immunity. Pharmacogenetic differences between individual patients and populations (affecting distribution, metabolism, and clearance) might also contribute to additional variation in LAmB efficacy in the clinic (20).

Finally, although BALB/c mice are common in PK studies (56) and L. major-BALB/c is a highly reproducible and well-established model for antileishmanial drug evaluation (57), differences between CL in humans (mostly self-curing lesions) and BALB/c mice (nonhealing lesions) (58) should be considered. Our studies used mice with relatively small (<1 cm), local, and uncomplicated CL lesions. Despite variation in the immunological nature of the skin inflammation, the phenomena of capillary leakiness, edema formation, and phagocyte infiltration occur in both mice and humans (59, 60). Thus, our findings could hold treatment implications for CL as well as for other inflammatory (skin) disorders. During preclinical evaluation of novel nanoparticles, a drug delivery strategy used for CL (61), the time of drug administration (relative to disease stage), and causative species are important factors that can affect both PK and PD. In the clinic, LAmB treatment outcomes in CL are already known to be related to the causative Leishmania species. A recent observational study in a group of travelers with (M)CL (20) reported differences in the therapeutic success rate of LAmB against L. infantum (78%), L. major (50%), and Leishmania Viannia subgenus species (28%). However, because L. mexicana was not included in this work, we cannot directly compare our results in mice to those in humans. In addition, early diagnosis and therapeutic intervention with LAmB could produce enhanced drug exposure in the skin lesion. No present clinical studies have reported on this. In contrast, early treatment of L. brasiliensis CL with intramuscular pentavalent antimonials was associated with a 5-fold increased risk of treatment failure (62, 63). Both the impact of parasite species and the age of the lesion in CL on PK and therapeutic efficacy of LAmB (and other antileishmanial drugs) deserve further investigation. Laboratory experiments could investigate outcomes of multidose treatments in alternative models of disease caused by additional Leishmania species and strains. The extrapolation of LCL results to the various types of complex CL is complicated by differences in histopathology (blood vessel destruction in advanced MCL [10]) and the nature and severity of the inflammatory response (balance TH1/TH2-type cellular immunity in local versus diffuse CL [3, 4]). Overall, it is clear that the immunohistopathology of CL has a profound impact on drug disposition of antileishmanial agents, both when administered topically (increased permeation through the damaged epidermis [64, 65]) and systemically (enhanced extravasation for liposomal and nonencapsulated drugs [21]).

In conclusion, our data indicate that the severity of inflammatory skin disease in CL could contribute to variable drug penetration in the target tissue and therapeutic efficacy of LAmB. The significant impact of local inflammation on PK and PK/PD is not only an important consideration for the development of new drugs and clinical dose regimens for the treatment of CL but also for other (infectious) diseases with an inflammatory component.

## MATERIALS AND METHODS

### Parasites, media, and drugs.

L. major MHOM/SA85/JISH118 and L. mexicana MNYC/BZ/62/M379 parasites were cultured in Schneider's insect medium (Sigma, UK) supplemented with 10% heat-inactivated fetal calf serum (HiFCS; Sigma UK). These were passaged each week at a 1:10 ratio of existing culture to fresh media in 25-ml culture flasks without a filter and incubated at 26°C. For infection of mice, stationary-phase parasites were centrifuged for 10 min at 2,100 rpm and 4°C. The supernatant was removed and the pellet resuspended in RPMI medium (Sigma, UK). Cell number was estimated by microscopic counting with a Neubauer hemocytometer. AmBisome (LAmB; Gilead, UK) was reconstituted with 12 ml sterile water (as per the manufacturer's instructions) to yield a stock solution of 4 mg/ml and diluted in 5% aqueous dextrose to achieve a drug dose of 25 mg/kg. Paromomycin sulfate (Sigma) was prepared in phosphate-buffered saline (PBS) to yield 50-mg/kg doses. Lambda carrageenan (Sigma) and Evans blue (Sigma) 0.5% (wt/vol) solutions were made up in phosphate-buffered saline (PBS; Sigma). The drug preparations were stored at 4°C during the experiments.

### Experimental groups.

Female BALB/c mice around 6 to 8 weeks old and with a mean weight of 18 to 20 g were purchased from Charles River Ltd. (Margate, UK). These were kept in humidity- and temperature-controlled rooms (55 to 65% and 25 to 26°C, respectively) and fed water and rodent food *ad libitum*. Mice were randomized and allowed an acclimatization time of 1 week. All animal experiments were conducted under license 70/8427 according to UK Home Office regulations under the Animals (Scientific Procedures) Act 1986 and EC Directive 2010/63/E. An overview of the groups is shown in Fig. 1.

Group 1 was the L. major CL group. Mice were subcutaneously (s.c.) infected in the shaven rump above the tail with 200 μl of a parasite suspension containing 4 × 10^7^ of low-passage-number (<5), stationary-phase L. major promastigotes in RPMI medium. Lesion size was measured daily with digital calipers (average of length and width) after inoculation as the CL lesions developed into papules, initial nodules, and established nodules. In this animal model of CL, these respective disease stages occurred on days 5, 10, and 20, as shown previously (40). We define a CL lesion as a stationary local skin abnormality at the site of Leishmania parasite inoculation (rump). A “papule” is the smallest (2 to 4 mm) CL lesion, a palpable elevation of the skin with no signs of ulceration. An “initial nodule” is a medium-sized (4 to 6 mm) papule that is larger and more defined. An “established nodule” is a larger (5 to 8 mm) CL lesion that is crusted or exudative.

Group 2 was the L. mexicana CL group. Mice were infected as described above for L. major, but L. mexicana promastigotes were used. In this animal model of CL, the disease stages of papule, initial nodule, and established nodule occurred on days 15, 30, and 45 postinoculation (40). The above-described definitions of CL lesion, papule, initial nodule, and established nodule apply.

Group 3 was skin inflammation controls. For the uninfected controls, mice were infected in the shaven rump above the tail with 200 μl parasite-free RPMI medium (s.c.). For the healed lesion (HL) controls, mice with L. major initial nodules (10 days postinoculation, infection as described above) were treated daily for 10 days with 50 mg/kg paromomycin sulfate in PBS (200 μl via the intraperitoneal [i.p.] route). This regimen has proven efficacy in the L. major-BALB/c model of CL (40). A size of 0 mm (complete disappearance of the skin lesion) was considered a near-complete healing and a negative control for skin inflammation. For the “pseudolesion” (PL) control, mice were s.c. injected in the shaven rump above the tail with 25 μl of 0.5% λ carrageenan in PBS. After 24 h, when a measurable lesion-like but parasite-free swelling of skin had occurred, the pseudolesion was considered a positive control for skin inflammation. These specific concentration and time points were chosen based on similarity to CL lesions and experimental requirements. The resulting diameter of the skin swelling (lesion size) was between 2 and 8 mm (the size of our CL lesions). Moreover, the local inflammation remained for at least 48 h (24 h to reach maximal swelling and another 24 h for the PK experiment). This novel carrageenan-induced model of local rump skin inflammation in mice was based on the well-established model of rat paw inflammation (38, 39), and preparatory studies are shown in the supplemental material.

### Procedures per experimental group.

Ten mice per group (L. major papule, L. major initial nodule, L. major established nodule, L. mexicana papule, L. mexicana initial nodule, L. mexicana established nodule, uninfected, pseudolesion, and healed lesion) were divided in a pharmacokinetic (*n* = 5) and skin pathophysiology arm (*n* = 5). This allowed simultaneous studying of drug accumulation 24 h after LAmB administration (this time point results in maximal AmB accumulation in skin [21]) and pathophysiology factors affecting pharmacokinetics at the time of drug administration (30 min after administration of Evans blue, standard time for preferential distribution of the dye to inflamed compared to healthy peripheral tissue sites [35–37]). An overview of the procedures performed per group is shown in Fig. 1.

### (i) Pharmacokinetic arm.

Each animal in this arm (*n* = 5) received an i.v. bolus (200 μl) of LAmB at a dose level of 25 mg/kg. Twenty-four hours later, animals were sacrificed, and skin samples (from lesion and healthy control site) were collected. The skin samples were homogenized and AmB levels in tissues measured as previously described (21, 33). Briefly, skin tissues were ground mechanically with zirconium oxide beads in 1 ml of PBS. The drug (AmB) was then extracted from tissue homogenates with 84:16 methanol-dimethyl sulfoxide (methanol-DMSO), followed by liquid chromatography-tandem mass spectrometry (LC-MS/MS) quantification. When the expression “AmB levels” or “AmB concentrations” is used in this work without further clarification, it refers to total (liposomal + protein-bound + free) amount of AmB per gram of tissue. Pharmidex Pharmaceutical Services Ltd. performed LC-MS/MS analysis of the samples. The lower limit of quantification was 1 ng/ml.

### (ii) Skin pathophysiology arm.

Each animal in this arm (*n* = 5) received an intravenous bolus (200 μl) of 0.5% Evans blue (Sigma, UK). Lesion size (average of width and length in millimeters) was measured with digital calipers. Thirty minutes later, animals were sacrificed, and skin samples (from the lesion and the healthy control site) were collected. These samples were cut into three equal parts, weighed, and used for the evaluations described below.

### Capillary leakiness.

The first skin fragment was used to evaluate blood vessel leakiness with the Evans blue assay. Evans blue is a blue dye which is, under normal physiological conditions, predominantly restricted to the bloodstream because of high plasma protein binding. However, the protein-dye complex can extravasate at sites of increased vessel leakiness, as is the case in local inflammation. Hence, the amount of Evans blue per gram of tissue is a marker for local vascular permeability (35–37). To extract Evans blue from the skin, tissue sections were placed in 500 μl formamide in Eppendorf tubes and incubated in a 55°C water bath. After 24 h, tubes were centrifuged for 10 min at 15,000 rpm and 4°C, and supernatants were collected. Absorbance (maximum, 620 nm; minimum, 740 nm) was determined with a SpectraMax M3 plate reader (Molecular Devices, UK). Samples, blanks (formamide), and calibration standards (1:2 serial dilution of 100 μg/ml Evans blue in formamide) were measured in 96-well plates (200 μl volumes). After correction against the blank, the amount of Evans blue in samples was expressed per gram of skin tissue.

### Parasite load.

The second skin tissue fragment was used to evaluate L. major and L. mexicana parasite loads with DNA-based quantitative PCR, as described previously (40). In brief, skin tissue was homogenized and DNA extracted with a Qiagen DNeasy blood and tissue kit. Two-microliter DNA extract samples (1/100 diluted) were amplified in 10-μl reaction mixtures in the presence of 5 μl SensiFAST SYBR NO-ROX master mix, 0.25 μM probe, and 0.4 μM primers. Triplicates of standards (10^8^ to 10^2^) and duplicates of unknown samples were included. The tubes were placed in a 72-sample rotor of the Rotor-Gene 3000, set at 40 cycles at a denaturation setting of 95°C for 5 min, followed by a 2-step amplification cycle of 95°C for 10 s and 60°C for 30 s. The lower limit of quantification was 100 parasites per 2 μl.

### Skin histomorphometry.

The third and final skin fragment was fixed in formalin for 24 h, dehydrated in ethanol, cleared in xylene, and embedded in paraffin. Skin samples were stained with hematoxylin and eosin (H&E) or antibodies against the macrophage/microglia-specific protein iba-1 (anti-Iba-1). All histological procedures were performed at the Institute of Neurology (UCL, London, UK), and blind analysis using the same analyst was conducted at LSHTM. A Leica ST5020 Autostainer was used for H&E staining, according to the standard National Health Service (NHS) diagnostic protocol. Randomly selected images covering skin regions were acquired with a camera (Leica DFC295) attached to a Leica DM3000 light-emitting diode (LED) microscope. Images were digitalized for histomorphometric analysis using the Leica Application Suite V4.5 software. An index of inflammatory cells was assessed by quantifying a standardized test area of 166,970.7 μm^2^ per image acquired, with a ×20 objective. The number of cells per image was determined from the average of 6 images/animal, randomly chosen, at ×200 magnification, stained with H&E. An increase in the number of cells compared with uninfected controls was considered indicative of inflammation. Immunohistochemistry reaction for macrophage presence was performed using the Ventana Discovery XT using the Ventana DAB map detection kit. Tissues were pretreated for 40 min with EDTA buffer, incubated for 4 h with the primary antibody (anti-Iba-1, 1/250 dilution; Wako Laboratory Chemicals, Germany), and treated with swine anti-rabbit Dako E0353 antibody for 1 h (manufacturer's protocol). The polyclonal antibodies in the anti-Iba-1 stain label the calcium-binding protein Iba-1, specific to microglia (central nervous system) and macrophages (skin and other tissues). An index of macrophage was assessed by quantifying a standardized test area of 166,970.7 μm^2^ per image, acquired with a ×20 objective. The area in brown was determined from an average of 6 randomly chosen images/animal, at ×200 magnification. Increased stained area compared with uninfected controls was considered indicative of macrophage infiltration.

### Efficacy of LAmB against L. major and L. mexicana.

Uninfected or Leishmania-infected BALB/c mice with nodular CL lesions (10 and 30 days postinoculation for L. major and L. mexicana, respectively) received five doses (200 μl, i.v.) of either 5% dextrose (untreated control) or LAmB at 25 mg/kg (treated) on alternate days (i.e., on days 0, 2, 4, 6, and 8). During treatment, lesion size was monitored daily. On day 10, animals were sacrificed, lesion samples were collected, and parasite load and AmB drug levels in these tissues were quantified (see above).

### Statistical analysis.

For the PK and pathophysiology experiments, intralesional AmB accumulation, lesion size, parasite load, capillary leakiness, cell number, and macrophage abundance were compared in infected and uninfected skin of the same mice using a 2-way analysis of variance (ANOVA), followed by a Šidák multiple-comparison test. For the efficacy experiment, ANOVA (1-way for parasite load and intralesional AmB levels, 2-way repeated measures for lesion size) followed by Tukey's multiple-comparison test were used. Data are presented as mean and standard error of the mean (SEM). A *P* value of <0.05 was considered statistically significant. All analyses were performed with GraphPad Prism version 7.02.

## Supplementary Material

Supplemental file 1
