# Supplementary material for: Local Skin Inflammation in Cutaneous Leishmaniasis as a Source of Variable Pharmacokinetics and Therapeutic Efficacy of Liposomal Amphotericin B
Source: Antimicrob Agents Chemother. 2018 Sep 24;62(10):e00631-18. doi: 10.1128/AAC.00631-18 (PMC6153808; doi:10.1128/AAC.00631-18)
Supplement: Supplemental file 1 [file zac010187540s1.pdf]

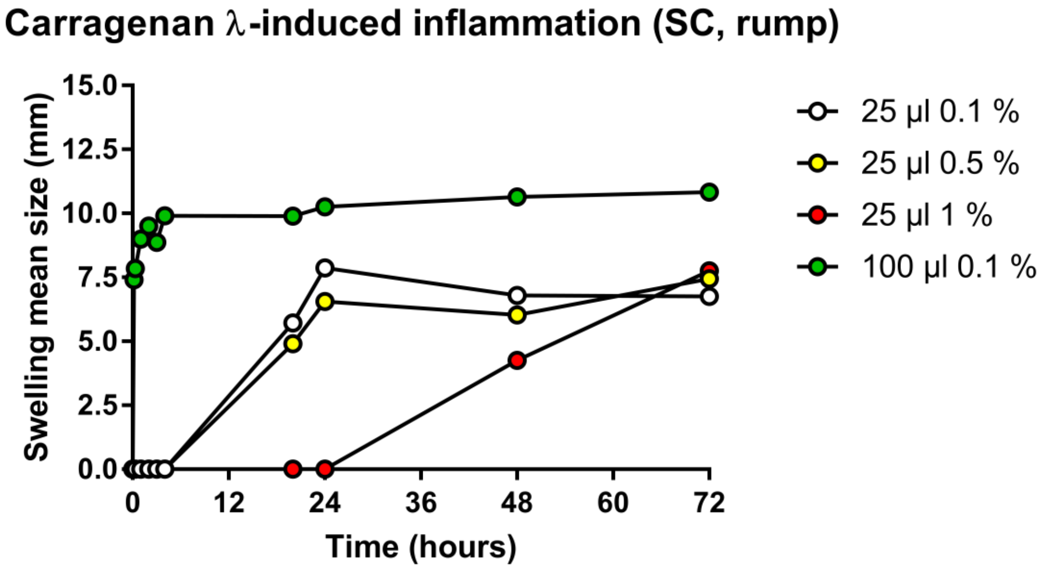

Supplementary figure 1: Testing of carrageenan-λ as a suitable agent to create a “pseudolesion”, a CL-like but parasite-free nodule and positive control for skin inflammation. Different volumes and concentrations were used for subcutaneous injection in the rump. The aim was to obtain a “lesion size” was between 2-8 mm (the size of our CL lesions), and a local inflammation type that remained for at least 48 hours (24 hours to reach maximal swelling and another 24 hours for PK experiment).
